# Breeding of a new wastewater treatment yeast by genetic engineering

**DOI:** 10.1186/2191-0855-1-7

**Published:** 2011-05-25

**Authors:** Miyoshi Kato, Haruyuki Iefuji

**Affiliations:** 1 Graduate School of Biosphere Science, Hiroshima University, 1-4-4, Kagamiyama, Higashihiroshima, Hiroshima 739-8527, Japan; 2 National Research Institute of Brewing, 3-7-1, Kagamiyama, Higashihiroshima, Hiroshima 739-0046, Japan

## Abstract

We previously developed a host vector system for the wastewater treatment yeast *Hansenula fabianii *J640. The promoter and terminator regions of the gene encoding glucoamylase from *H. fabianii *J640 were used for a new expression vector, pHFGE-1. The performance of pHFGE-1 was compared with that of the widely used pG-1 transformant vector. *H. fabianii *J640 (HF-TAMY) cells were transformed with pHFGE-1, and *Saccharomyces cerevisiae *YPH-499 (SC-TAMY) cells were transformed with pG-1, both of which carried the Taka-amylase. Expression of Taka-amylase by HF-TAMY showed higher than that by SC-TAMY. By using this new system, we bred the new wastewater treatment yeast that shows α-amylase activity. This yeast appears to grow well under experimental wastewater conditions, and is effective in treating model wastewater containing soluble and insoluble starch.

## Introduction

Many food factories use wastewater treatment systems that use yeasts (Yoshizawa 1978, 1981, Sato et al. 1986, Moriya et al. 1990, Suzuki et al. 1991, Suzuki et al. 1996). However, some wastewater-containing polysaccharides, such as raw starch and hemicellulose, are difficult to treat because presently used yeasts secrete few enzymes that can digest these polysaccharides. One way to treat these wastewaters is to transform conventional wastewater treatment yeasts with the genes for polysaccharide-digesting enzymes such as raw starch-digesting α-amylase and acid xylanase.

To this end, we isolated *Cryptococcus *sp. S-2 (Iefuji et al. 1994), which secretes several enzymes including raw starch-digesting α-amylase (Iefuji et al. 1996a), acid xylanase (Iefuji et al. 1996b), lipase (Kamini et al. 2000) and polygalacturonase. We then obtained the genes that encode the raw starch-digesting α-amylase and acid xylanase.

*H. fabianii *J640 is a commonly used wastewater treatment yeast (Saito et al. 1987, Sato et al. 1987, Suzuki et al. 1996). We previously constructed an expression system based on this strain (Kato et al. 1997). A uracil auxotrophic mutant of *H. fabianii *J640, named *H. fabianii *J640 u-1, lacking orotidin-5'-phosphate decarboxylase, was obtained. We constructed a plasmid, pHFura3, that contains the gene encoding orotidine-5'-phosphate decarboxylase of *H. fabianii *J640. In the previous study (Kato et al. 1997), by employing *H. fabianii *J640 u-1 as a host strain and pHFura3 as a vector plasmid, we constructed a transformation system of *H. fabianii *J640.

We purified the glucoamylase of *H. fabianii *J640 and cloned its cDNA and genomic DNA (Kato et al. in press). Then, we constructed a new expression vector, pHFGE-1 (Kato et al. in press), which uses pHFura3, and the promoter and terminator regions of the gene encoding glucoamylase from *H. fabianii *J640. We inserted the genes encoding α-amylase and xylanase from *Cryptococcus *sp. S-2 between the promoter and terminator of pHFGE-1. When the pHFGE-1 with one or the other of these foreign genes were transformed into *H. fabianii *J640 u-1, the transformants (named HF-AAMY and HF-XYN, respectively) showed α-amylase and xylanase activities respectively. This showed that pHFGE-1 can derive the expression of foreign genes in *H. fabianii *J640 cells.

In this paper, we investigated the ability of these transformed yeasts, to treat wastewater, and developed a PCR method for monitoring the presence of the foreign gene.

## Materials and methods

### Strains and media

Strains *H. fabianii *J640 and *Cryptococcus *sp. S-2 were obtained from the National Research Institute of Brewing culture collection, Japan. A uracil auxotrophic mutant of *H. fabianii *J640, named *H. fabianii *J640 u-1, lacking orotidine-5'-phosphate decarboxylase, was used as a host strain for new expression vector pHFGE-1. *S. cerevisiae *YPH-499 (MATα ura3 lus2 ade2 trp1 his3 leu2) was used as the host for transformation vector pG-1 (Schena et al. 1991). *E. coli *strain HB101 and JM109 were employed as the host of plasmid vector, which were used for DNA manipulation and construction of the gene library.

Yeast cells were grown on YM medium (0.3% yeast extract, 0.3% malt extract, 0.5% peptone and 1% glucose) and YPD medium (1% yeast extract, 2% peptone, 2% glucose). Luria-Bertani medium containing ampicillin (100 μg/ml) was used to cultivate *E. coli*. The minimal medium containing 1% glucose and 0.67% yeast nitrogen base (YNB) without amino acids was used to select the yeast transformants. YPM medium was prepared by replacing the glucose of YPD with maltose. The medium used to investigate expression induction, contained 1% yeast extract, 1% casamino acid, and 2% glucose or maltose.

### Expression vector for *H. fabianii *J640

**Figure 1:**
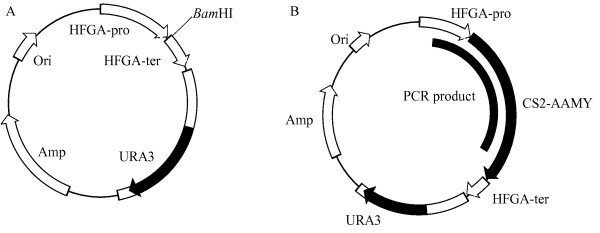
**Restriction map**. (A) Expression vector pHFGE-1. (B) Position of PCR product in pHFGE-AMY for monitoring (black arc inside circle). CS2-AAMY, α-amylase gene from *Cryptococcus *sp. S-2

The expression vector pHFGE-1 (Kato et al. in press) (Figure 1A) was used. The cloning site of this vector is a *Bam*HI site between the promoter and terminator from *H. fabianii *J640 glucoamylase DNA. The host cell of this vector is a uracil auxotrophic mutant designated as *H. fabianii *J640 u-1, and it could be transformed by a non-homologous and frequently multicopy integration into the host genomic DNA.

### Transformation of yeast

Transformations were carried out by electroporation as described by (Becker et al. 1991). Electroporation was done with a Gene Pulser (Bio-Rad) with settings of 200 V and 25 μF using a 0.2 cm cuvette.

### Assay of xylanase and α-amylase activity

Xylanase activity was assayed by measuring the amount of reducing sugar liberated from xylan (Iefuji et al. 1996b). One unit of activity was defined as the amount of xylanase needed to liberate 1 μmol of D-xylose per min under the condition just described.

α-Amylase activity was measured with an α-amylase kit (Kikkoman). One unit of α-amylase activity was defined as the amount of enzyme which forms 1 μmol of 2-choloro-4-nitrophenol from 2-choloro-4-nitrophenyl 6^5^-azide-6^5^-deoxy-β- maltopentaoside under the condition described above.

### Preparation of model wastewater and treatment test

Model wastewater containing soluble starch was made with 1% refined starch (Merck) and 0.25% yeast extract, pH 6.0. The starch was solubilized by autoclaving. Model wastewater containing insoluble starch was made with 0.25% yeast extract, pH6.0, autoclaved and cooled to approximately 55°C. The same amount of starch was sterilized in 70% ethanol. The suspension was centrifuged and decanted. The starch pellet was then added to the autoclaved yeast extract solution.

Yeast cells were incubated at 30°C for 2 days on YM medium. Then 5 × 10^6 ^cells/ml was inoculated to the model wastewater in an Erlenmeyer flask. Cultures were incubated at 30°C with shaking at 105 rpm and samples were periodically harvested.

### Yeast cells in the model wastewater were counted with a hemocytometer

The model wastewater containing soluble starch was centrifuged at 3000 rpm for 10 min, and chemical oxygen demand (COD) of the supernatant was measured. The decrease in COD of the model wastewater containing soluble starch was used to express the capacity of the yeast to treat the wastewater.

It was not possible to measure COD of the model wastewater containing insoluble starch because of the difficulty in separating the cells and insoluble starch. In this case, degradation of the starch was measured with the iodo-starch reaction (Sato et al. 1987) as follows: 1 ml culture was heated in a micro tube at 100°C for 30 min to solubilize the starch. Yeast cells were then removed by centrifugation. Iodic liquid (0.2 ml; containing 0.0317 g iodine, 0.1 g potassium iodide and 5 ml 3N-HCl in 100 ml water) was added to the supernatant and the optical density was measured at 670 nm. Transmittance at 670 nm was taken as a measure of starch degradation.

### Monitoring the presence of a foreign gene in a transformant

The transformants were cultured in 10 ml YM medium and harvested by centrifugation. DNA was extracted with an Easy-DNA kit (Invitrogen) and used for the PCR template. Unique PCR primers were designed, and the position of the PCR product is shown in Figure 1B. PCR cycling conditions were followed by 25 cycles of 94°C for 1 min, 55°C for 2 min, 72°C for 3 min.

To determine the sensitivity of the PCR, cells were cultured in YM medium, and the cell density was measured. Then a dilution series was made (10^6^-10^1 ^cells/ml). One ml of each dilution was harvested and DNA was extracted with the EASY-DNA kit and used as a PCR template.

## Results

### Induction of foreign gene expression

Glucoamylase production by *H. fabianii *J640 was induced by maltose and repressed by glucose. Since our constructed expression vector used the promoter and terminator regions of the *H. fabianii *J640 glucoamylase gene, we expected that foreign gene expression in the transformant would also be induced by maltose. As expected, xylanase production by HF-XYN was highest, when maltose was the C source with yeast extract and casamino acid as media components (Table 1).

**Table 1:** Effect of media components on xylanase activity

Media composition	Xylanase activity (U/ml)
Maltose, YNB w/o amino acids	37

Maltose, Yeast extract, Casamino acid	310

Glucose, YNB w/o amino acids	1

Glucose, Yeast extract, Casamino acid	6

### Comparison of two vectors

**Figure 2:**
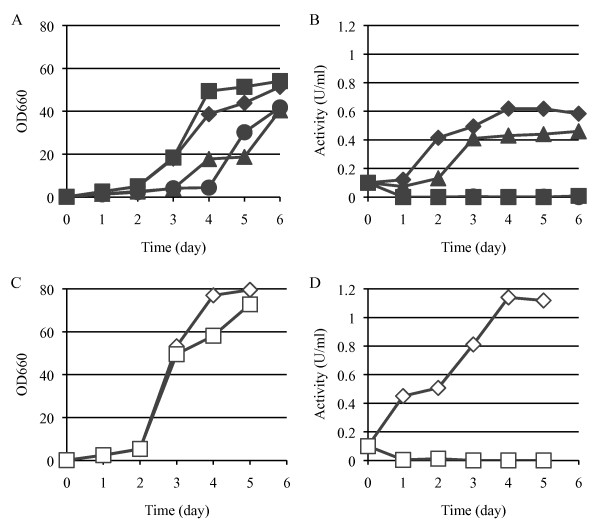
**Comparison of two vectors and carbon sources**. (A) Growth of transformants on YPD medium. (B) α-Amylase activity of transformant on YPD medium. (C) Growth of transformants on YPM medium. (D) α-Amylase activity of transformant on YPM medium. The strains are: HF-TAMY (diamonds), transformant with pHFGE-1 (not connected to any gene), into *H. fabianii *J640 u-1 (squares), SC-TAMY (triangles), transformant with pG-1 (not connected to any gene), into *S. cerevisiae *YPH-499 (circles).

The performance of our expression vector pHFGE-1 was compared with that of the widely used pG-1 transformation vector. *H. fabianii *J640 u-1 (HF-TAMY) cells were transformed with pHFGE-1, and *S. cerevisiae *YPH-499 (SC-TAMY) cells were transformed with pG-1, both of which carried the Taka-amylase gene. The cells were then cultured on YPD and YPM media. Growth on YPD medium was the same for the two cultures (Figure 2A). α-Amylase activity was a little higher in the HF-TAMY cells than in the SC-TAMY (Figure 2B). The α-amylase activity of HF-TAMY cells was highest when maltose was the C source (YPM medium, Figure 2D), indicating that gene expression was induced by maltose.

### Treatment of model wastewater

**Figure 3:**
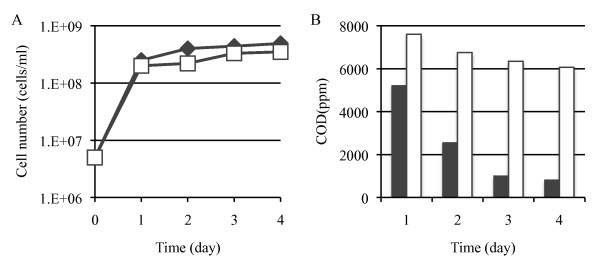
**Treatment test of model wastewater containing soluble starch**. (A) Growth rate of cells. (B) Decrease of COD. The strains are: HF-AAMY (diamonds, black bars), *H. fabianii *J640 (host strain) (squares, white bars).

**Figure 4:**
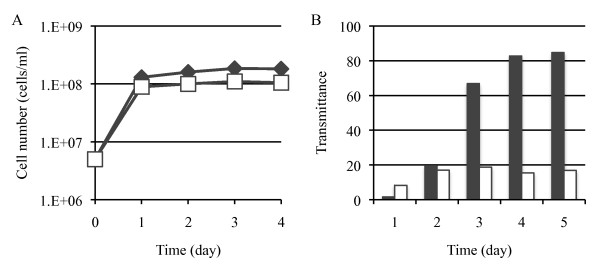
**Treatment test of model wastewater containing insoluble starch**. (A) Growth rate of cells. (B) Resolution capacity of the insoluble starch. The strains are: HF-AAMY (diamonds, black bars), *H. fabianii *J640 (host strain) (squares, white bars).

HF-AAMY cells and cells of the parent strain *H. fabianii *J640 grew at about the same rate in the model wastewater containing soluble starch or insoluble starch (Figure 3A or 4A). Both the parent strain and HF-AAMY decreased the COD of the model wastewater containing soluble starch to decrease, although the decrease was much faster with the HF-AAMY cells (Figure 3B). The HF-AAMY cells were also much more efficient at degrading the insoluble starch (Figure 4B). These results indicate that HF-AAMY cells have a high capacity to treat wastewater containing starch.

### Monitoring of transformant by PCR

**Figure 5:**
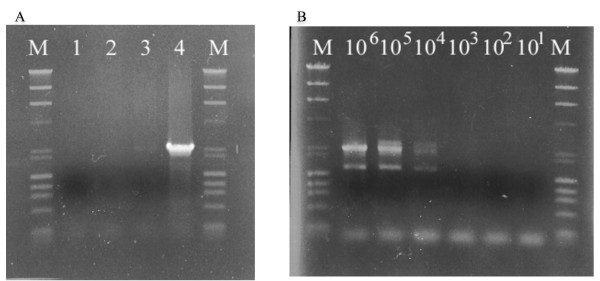
**PCR test**. (A) Specificity of PCR test for HF-AAMY cells. M, marker The strains are: (1) *S. cerevisiae *YPH-499, (2) *Cryptococcus *sp. S-2, (3) *H. fabianii *J640 (host strain), (4) HF-AAMY (transformant). (B) Sensitivity of PCR test. The number of cells in the reaction mixture are shown at the tops of the lanes.

Of four strains (*S. cerevisiae *YPH-499, *Cryptococcus *sp. S-2, *H. fabianii *J640 and HF-AAMY (the transformant)), only the transformant showed a PCR product (Figure 5A) corresponding to part of the HFGA promoter and the α-amylase gene (Figure 1B). The detection sensitivity of PCR which uses Taq Plus Long PCR kit (Stratagene) was high, i.e., it could detect only 10^4 ^cells (Figure 5B). The different intensities of the PCR bands in Figure 5B are presumably the result of the different cell densities in the cultures.

## Discussion

We developed a host vector system for the wastewater treatment yeast, *H. fabianii *J 640, and we created new wastewater treatment yeast transformants (HF-XYN and HF-AAMY). The expression of the foreign gene that was integrated in the transformant was induced by maltose and repressed by glucose. However, the growth rates of the transformants carrying pHFGE-1 and the widely used pG-1 were the same and both transformants strongly expressed the foreign gene, even in medium containing glucose, which was expected to repress expression of the foreign gene. Our host vector system strongly expresses the foreign gene. Because wastewater contains various components, the strong expression of the new strain is an advantage. The HF-AAMY cells were effective in treating the model wastewater.

Because HF-AAMY cells are genetically modified, a sensitive method for monitoring the cells in the environment is needed. Our PCR was shown to satisfy this requirement.

A host vector system was also developed for the methylotrophic yeast *Hansenula polymorpha *(Gellissen et al. 2004, Steinborn et al. 2006). As in these systems, auxotrophic strains (ura-, leu-) were used as the host. The expression cassettes in these systems used the promoters for various genes, including the genes for formate dehydrogenase (FMD), methanol oxidase (MOX), and trehalose-6-phosphate synthase (TPS1). *H. polymorpha *is rapidly becoming the system of choice for heterologous gene expression in yeast. Several production processes for recombinant pharmaceuticals and industrial enzymes have been developed based on gene expression in this strain. Another methylotrophic yeast, *Hansenula ofunaensis*, has also been evaluated for a transformation system (Yamada-Onodera et al. 1999, Yamada-Onodera et al. 2006) but development has not been completed.

A transformation system using *Hansenula anomala*, another wastewater treatment yeast, was developed in the 1990s (Ogata et al. 1992, Ogata et al. 1995). However, none of these studies of wastewater treatment yeasts constructed an expression vector or bred new strains of yeast. With the new transformation system, it should be possible to treat wastewater containing polysaccharides that are presently resistant to degradation.

Our next goal is to use our transformant to treat real wastewater from the food industry. In the future, when genetically engineered yeast is proven to be effective for the treatment of wastewater, a major task will be to prove to the public that the methodology is safe.

## Competing interests

The authors declare that they have no competing interests.
